# A novel hybrid organosolv: steam explosion method for the efficient fractionation and pretreatment of birch biomass

**DOI:** 10.1186/s13068-018-1163-3

**Published:** 2018-06-08

**Authors:** Leonidas Matsakas, Christos Nitsos, Vijayendran Raghavendran, Olga Yakimenko, Gustav Persson, Eva Olsson, Ulrika Rova, Lisbeth Olsson, Paul Christakopoulos

**Affiliations:** 1 Biochemical Process Engineering, Division of Chemical Engineering, Department of Civil, Environmental, and Natural Resources Engineering, Luleå University of Technology, 971-87 Luleå, Sweden; 2 Division of Industrial Biotechnology, Department of Biology and Biological Engineering, Chalmers University of Technology, Kemivägen 10, 412 96 Göteborg, Sweden; 3 Department of Physics, Chalmers University of Technology, Fysikgränd 3, 412 96 Göteborg, Sweden; 4 Present Address: Department of Molecular Biology and Biotechnology, University of Sheffield, Sheffield, S10 2TN UK

**Keywords:** Fractionation, Cellulose-enriched biomass, Hybrid organosolv-steam explosion, Birch, Ethanol, High-gravity, Inhibitor-free biomass, Delignification, Cellic CTec2

## Abstract

**Background:**

The main role of pretreatment is to reduce the natural biomass recalcitrance and thus enhance saccharification yield. A further prerequisite for efficient utilization of all biomass components is their efficient fractionation into well-defined process streams. Currently available pretreatment methods only partially fulfill these criteria. Steam explosion, for example, excels as a pretreatment method but has limited potential for fractionation, whereas organosolv is excellent for delignification but offers poor biomass deconstruction.

**Results:**

In this article, a hybrid method combining the cooking and fractionation of conventional organosolv pretreatment with the implementation of an explosive discharge of the cooking mixture at the end of pretreatment was developed. The effects of various pretreatment parameters (ethanol content, duration, and addition of sulfuric acid) were evaluated. Pretreatment of birch at 200 °C with 60% v/v ethanol and 1% w/w_biomass_ H_2_SO_4_ was proven to be the most efficient pretreatment condition yielding pretreated solids with 77.9% w/w cellulose, 8.9% w/w hemicellulose, and 7.0 w/w lignin content. Under these conditions, high delignification of 86.2% was demonstrated. The recovered lignin was of high purity, with cellulose and hemicellulose contents not exceeding 0.31 and 3.25% w/w, respectively, and ash to be < 0.17% w/w in all cases, making it suitable for various applications. The pretreated solids presented high saccharification yields, reaching 68% at low enzyme load (6 FPU/g) and complete saccharification at high enzyme load (22.5 FPU/g). Finally, simultaneous saccharification and fermentation (SSF) at 20% w/w solids yielded an ethanol titer of 80 g/L after 192 h, corresponding to 90% of the theoretical maximum.

**Conclusions:**

The novel hybrid method developed in this study allowed for the efficient fractionation of birch biomass and production of pretreated solids with high cellulose and low lignin contents. Moreover, the explosive discharge at the end of pretreatment had a positive effect on enzymatic saccharification, resulting in high hydrolyzability of the pretreated solids and elevated ethanol titers in the following high-gravity SSF. To the best of our knowledge, the ethanol concentration obtained with this method is the highest so far for birch biomass.

## Background

Valorization of lignocellulosic biomass from forestry, agricultural, or other industrial side streams for the production of energy, chemicals, and materials, has been the subject of intensive research over the past decades [1]. This interest is based on the fact that lignocellulose is an abundant, renewable, and sustainable resource that can be used as raw material in environmentally friendly and economically beneficial processes. The technologies available for the utilization of lignocellulose are dictated by its chemical composition and structure. With a composition of as high as 70% sugars in the form of cellulose and hemicellulose polymers [2, 3], lignocellulose represents the feedstock of a glucocentric biorefinery process, which was focused initially on production of bioethanol via fermentation of the glucose fraction. The natural recalcitrance of lignocellulose to enzymatic degradation has led to the development of pretreatment strategies that disrupt its complex structure allowing an increased saccharification yield [4]. A number of acidic, aqueous-based pretreatment methods, such as steam explosion [5], dilute acid [6], and hydrothermal [7] have been evaluated toward this direction. The primary goal of these methods is to remove the hemicellulosic barrier around cellulose, while also partly disrupting the lignocellulosic structure, in order to reduce biomass resistance to enzymatic saccharification. Steam explosion causes a dramatic disruption of biomass structure with immediate reduction of particle size and defibration of the substrate [8]. These physical effects, combined with the removal of hemicellulose, lead to enhanced enzymatic saccharification of even the toughest substrates such as softwood-derived biomass [9]. Consequently, steam explosion has been considered for many years as a state-of-the-art pretreatment method in bioethanol production. However, such glucocentric strategy has been marred by a combination of high process costs—particularly regarding the production of cellulolytic enzymes [10]—and relatively small profit margins afforded by bioethanol [11]. To improve profitability, the hemicellulosic sugar fraction has been used as a feedstock for cofermentation with cellulose, and hence to increase the overall ethanol yield [12]. At the same time, lignin—the third polymeric component of lignocellulosic biomass—has been utilized as a low-cost fuel for generating heat or electricity and thus bringing down the overall cost of the process [13]. A more resource-efficient approach would be to utilize the entire biomass in a biorefinery concept, where the different process streams can be directed toward a wide range of products [14]. In this view, all lignocellulose components are potential sources of value-added products.

Implementation of biorefinery concepts depends greatly on the efficiency of the fractionation technologies used to separate cellulose, hemicelluloses, and lignin, and how well-defined the resulting streams are [11]. This has led to a shift regarding the role of lignin. In typical second-generation bioethanol production processes, lignin has been collected as a low-value byproduct and used for cogeneration of heat or electricity. However, when targeting added values from all biomass components, production of a high-purity lignin stream becomes a new necessity; especially since the phenolic components of lignin have been identified as a platform for the production of a variety of chemicals and polymers [15]. A comprehensive strategy for isolating lignin in the first step of the pretreatment/fractionation process is paramount to achieve a high-purity lignin-stream [16]. The requirement for ‘lignin-first’ removal is enhanced by the fact that lignin negatively affects the enzymatic saccharification of cellulose. This negative influence is the result of irreversible adsorption of cellulolytic enzymes onto lignin, and causes physical blockage of the enzymes on cellulose chains, as well as the inhibition of cellulolytic enzymes by soluble lignin-derived molecules [17]. Therefore, lignin removal during the first process step does not only provide a cleaner lignin stream, but can also improve the economics of traditional fermentation-based bioethanol processes as lignin can be used in high-value-added applications [18]. Organosolv pretreatment/fractionation represents one of the most promising biomass delignification and fractionation methods within the biofuels and biorefinery context [19, 20]. In the organosolv pretreatment/fractionation, biomass is heated up to a temperature range of 100–250 °C in an aqueous-organic solvent solution [21] for a specified duration resulting in three fractions: a solid dry lignin, an aqueous hemicellulose fraction, and a cellulose-rich solid fraction [22]. Low molecular weight aliphatic alcohols, such as ethanol, are frequently used as the organic solvent as they are easy to be recovered by distillation at the end of the organosolv and re-used in subsequent treatments [18, 23]. Implementation of such pretreatment/fractionation technologies is expected to facilitate the coexistence of traditional fermentation-based technologies with novel processes for the utilization of hemicellulose and lignin in broader biorefinery concepts, thus allowing for a multitude of products and higher profit margins [11]. It was previously shown that organosolv treatment and steam explosion pretreatment could be combined in a sequential way for the pretreatment of wheat straw [24] and fescue [25]; however, this significantly increases the process complexity (e.g., multiple stages of heating/cooling cycles and increased total process time).

The main aim of the current study was to develop a novel pretreatment method allowing for efficient fractionation of lignocellulosic biomass into cellulose, hemicellulose, and lignin streams. At the same time, the improved pretreatment method should be able to allow for high enzymatic saccharification yields of the cellulose stream for use in biochemical conversion processes. To attain this goal, we combined the fractionation efficiency of conventional organosolv processes with the benefit of physical biomass size reduction achieved during steam explosion into a single stage process. The suggested process was performed in a horizontal design steam explosion reactor, modified to also operate as an organosolv cooking vessel, as shown previously [26]. The novel method was carried out with an explosive discharge of the reactor’s content after conventional ethanol organosolv cooking. Process parameters such as time, ethanol content, and the addition of acid catalyst were studied for the effective pretreatment and fractionation of a representative hardwood biomass (birch). The effect of the explosion step on enzymatic saccharification of cellulose was also investigated. Finally, the ability of the proposed method to produce a cellulose-rich solid fraction that could effectively be saccharified and used during a biochemical conversion method was tested during high-gravity ethanol fermentation.

## Results and discussion

### Evaluation of fractionation efficiency of the hybrid method

#### Effect on biomass solubilization and composition of the pretreated solids

In our previous work on conventional batch organosolv fractionation of biomass [19], a pretreatment temperature of 182 °C for 60 min resulted in a 69% removal of lignin from birch biomass. To improve the performance of the hybrid organosolv method, a higher pretreatment temperature, 200 °C, was selected, and the effects of cooking time, ethanol content, and sulfuric acid concentration in the pretreatment liquor, in the presence of the explosive discharge step, were investigated. The effects of these parameters on the overall solubilization of birch biomass, the composition of the pretreated solids, and the solubilization of the major biomass component (cellulose, hemicellulose, and lignin) are summarized in Table 1. The aim of pretreatment was to obtain a solid material with high cellulose content and low hemicellulose and lignin content.

**Table 1:** Composition of pretreated solids at different pretreatment conditions

Pretreatment conditions			Biomass solubilization (% of initial biomass)	Cellulose (% w/w)	Hemicellulose (% w/w)	Lignin (% w/w)
Ethanol effect	30 min	50% v/v	47.9	65.9 (1.2%)	15.1 (74.8%)	6.7 (81.2%)
		60% v/v	48.9	67.1 (1.3%)	21.0 (65.7%)	7.1 (80.5%)
		70% v/v	41.5	61.7 (0.0%)	21.9 (59.0%)	8.7 (72.6%)
Time effect	60% v/v	15 min	44.7	66.3 (0.0%)	22.0 (61.1%)	7.8 (77.0%)
		30 min	48.9	67.1 (1.3%)	21.0 (65.7%)	7.1 (80.5%)
		60 min	40.0	60.7 (0.0%)	19.1 (63.4%)	13.2 (57.5%)
Catalyst effect	15 min—60% v/v ethanol	0%	44.7	66.3 (0.0%)	22.0 (61.1%)	7.8 (77.0%)
		0.2%	45.0	61.1 (3.2%)	25.8 (54.5%)	7.4 (78.2%)
		1%	63.1	77.9 (17.2%)	8.9 (89.5%)	7.0 (86.2%)

All results are expressed based on dry mass. Numbers in parenthesis represent the mass fraction of each component (i.e., cellulose, hemicellulose, and lignin) that was solubilized at the end of the pretreatment (calculated using Eq. 1). Compositional analysis was performed in duplicates, and the standard error was > 5% of the value. All the trials were done with the explosive discharge step at the end of the treatment

A reduction of ethanol content from 70 to 50% v/v during constant treatment led to an increase in biomass solubilization from 41.5 to 48–49% (calculated as the dry-mass fraction recovered as pretreated solids). This increase could be explained by the increased chemical hydrolysis of carbohydrates, and possible cleavage of α- and β-ether bonds of lignin [27], caused by the higher water content and its increased chemical activity in the pretreatment liquor. Reduced ethanol content in the pretreatment liquor also had a positive effect on cellulose level, which increased from 61.7% at 70% v/v ethanol to 65.9% at 50% ethanol and was accompanied by a reduction in hemicellulose content from 21.9 to 15.1%. Cellulose solubilization during pretreatment was not affected by the varying ethanol concentration employed (Table 1) and cellulose was completely recovered in the pretreated solid. Hemicellulose solubilization increased from 59 to 66 and 75% as ethanol decreased from 70 to 60 and 50%, respectively (Table 1). The latter had also a positive impact on delignification (Table 1). A similar trend had been observed during organosolv treatment of wheat straw, where delignification decreased from 38.8 to 20.8% when ethanol content increased from 50 to 80% w/w [28]. Increased delignification with decreasing ethanol content is a consequence of the higher chemical activity of water, resulting in the cleavage of ether linkages and concomitant lignin fragmentation [27, 29].

An ethanol content of 60% v/v was used to evaluate the effect of cooking time. Increasing cooking time from 15 to 30 min had a positive impact on biomass solubilization, whereas a further increase to 60 min had a negative effect (Table 1). Moreover, increasing the treatment time from 15–30 to 60 min led to a drop in cellulose content in the pretreated solids from 66–67 to 61% w/w, respectively (Table 1), even though no cellulose solubilization was observed during the pretreatment. Prolonged pretreatment time (60 min) resulted in a higher lignin content and a decreased delignification of the materials, possibly due to the formation of pseudo-lignin [30, 31], as well as lower hemicellulose content in the solid fraction and increased hemicellulose solubilization. Formation of insoluble lignin-like compounds or pseudo-lignin (mainly from the hemicellulose decomposition) is well documented in the literature, and these compounds are normally measured as lignin content, thus increasing the determined lignin content in the pretreated solids [32–34]. The decrease in solubilization under harsher pretreatment conditions could also be attributed to the formation of the pseudo-lignin.

Using the 15-min treatment as a reasonable compromise between time and efficiency, addition of an acidic catalyst (sulfuric acid) on pretreatment efficiency was examined. Addition of the catalyst at 0.2% w/w of biomass had almost no impact on the overall biomass solubilization, whereas a 1% w/w catalyst concentration increased biomass solubilization to 63%, mainly due to extensive cleavage of carbohydrates and aryl-ether bonds of lignin [35, 36]. Addition of 1% w/w acid catalyst resulted in very high cellulose content (78%) and increased solubilization of hemicellulose (Table 1), with part of the cellulose being solubilized due to the acid. In contrast, addition of 0.2% acid catalyst did not improve hemicellulose solubilization compared to pretreatment without catalyst. The same trend was observed for delignification: at the lower concentration the catalyst had only a minor positive impact, whereas at the higher concentration delignification increased from 77 to 86% (Table 1). The extended solubilization of hemicellulose and lignin is a result of the severe conditions during the pretreatment caused by the higher acid concentration.

In general, the novel pretreatment system resulted in pretreated solids with high cellulose content. Hemicellulose was resilient to all pretreatment conditions and its content in the pretreated solids was relatively high (Table 1). Indeed, with the exception of the high-concentration acid catalyst pretreatment (where 89.5% of the initial hemicellulose solubilized), the percentage of hemicellulose solubilization was between 54.5 and 74.8% of the initial hemicellulose fraction (Table 1). The main advantage of the proposed pretreatment method when using birch biomass was its effective lignin removal. Specifically, lignin content of the pretreated solids was below 9% in most cases, and dropped to 7% when 1% acid was used. Such highly efficient delignification, combined with elevated cellulose content in some pretreated solids, is very promising not only as a biomass fractionation method, but also for effective and low-cost enzymatic hydrolysis of pretreated solids [17]. In addition, high cellulose content is necessary to achieve high ethanol titers in bioethanol production.

#### Lignin purity

**Fig. 1:**
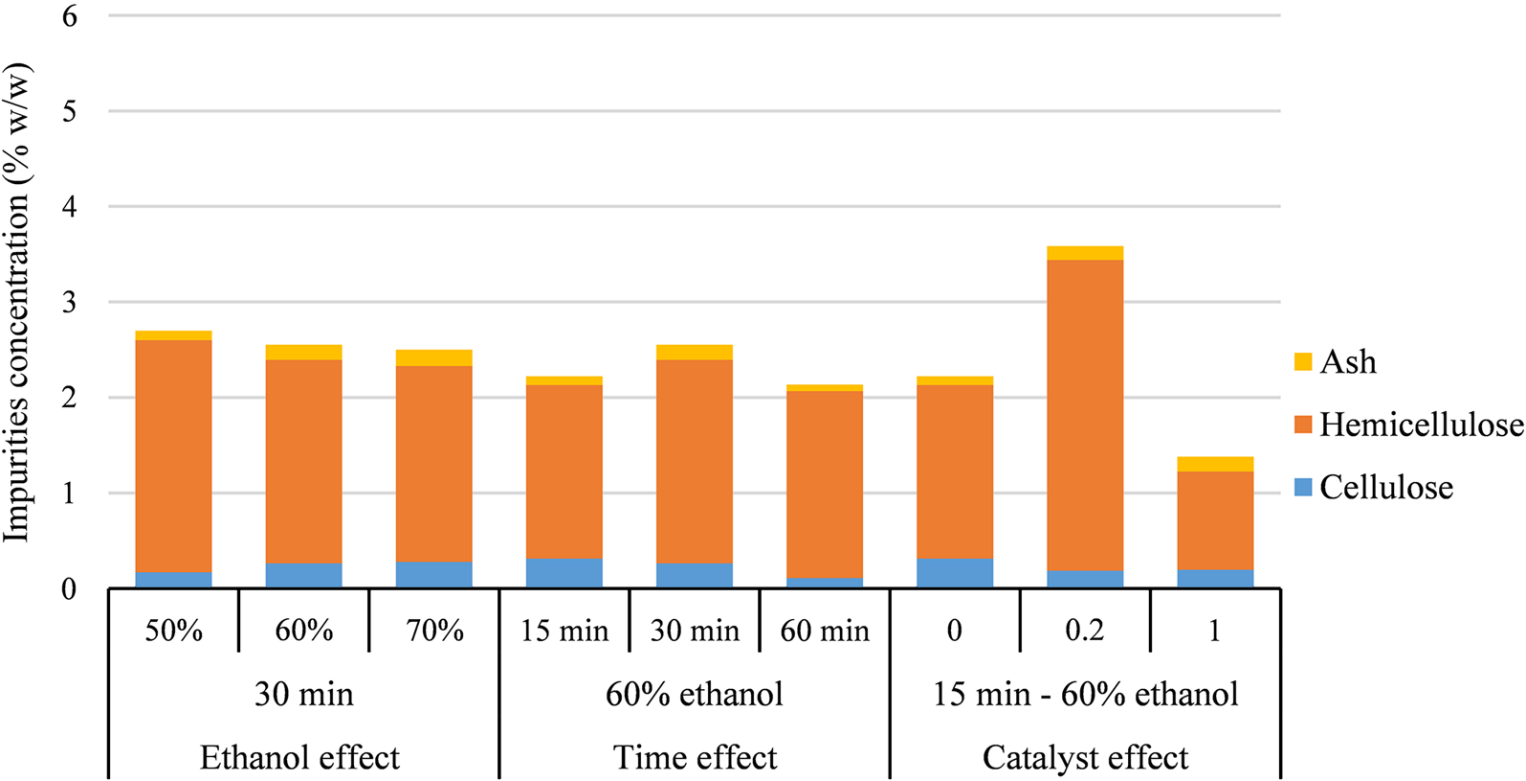
Carbohydrate and ash contents in the lignin fraction obtained under different treatment conditions with birch biomass. The analysis was done in duplicates

As discussed before, the hybrid pretreatment method developed here resulted in a process with delignification yields as high as 86% (as calculated with the Eq. 1). Besides the yield, the percentage of impurities (e.g., cellulosic and hemicellulosic sugars and ash) in the recovered lignin is also important when considering the utilization of lignin in a biorefinery to produce fuels, chemicals, or materials. The carbohydrate and inorganic ash contents of the different lignin fractions are presented in Fig. 1. Lignin purity remained high throughout the whole range of pretreatment conditions evaluated. Ash content was minimal and did not exceed 0.17% w/w; and the cellulose content remained low, between 0.11% and 0.31% w/w. Hemicellulose sugars were slightly higher, but they never exceeded 3.25%; the lowest carbohydrate content was obtained with 1% sulfuric acid. Overall, purity of > 96% was achieved in all samples, thus offering a very efficient fractionation of high-quality lignin. High purity and low ash content (especially low sulfur content) are unique qualities of organosolv lignin compared to Kraft pulping [37]. Due to the efficient delignification by the novel hybrid pretreatment method, lignin depositions on the biomass were not observed (see “Assessing the role of explosive discharge” section).

#### Sugar composition in the liquid fraction

**Fig. 2:**
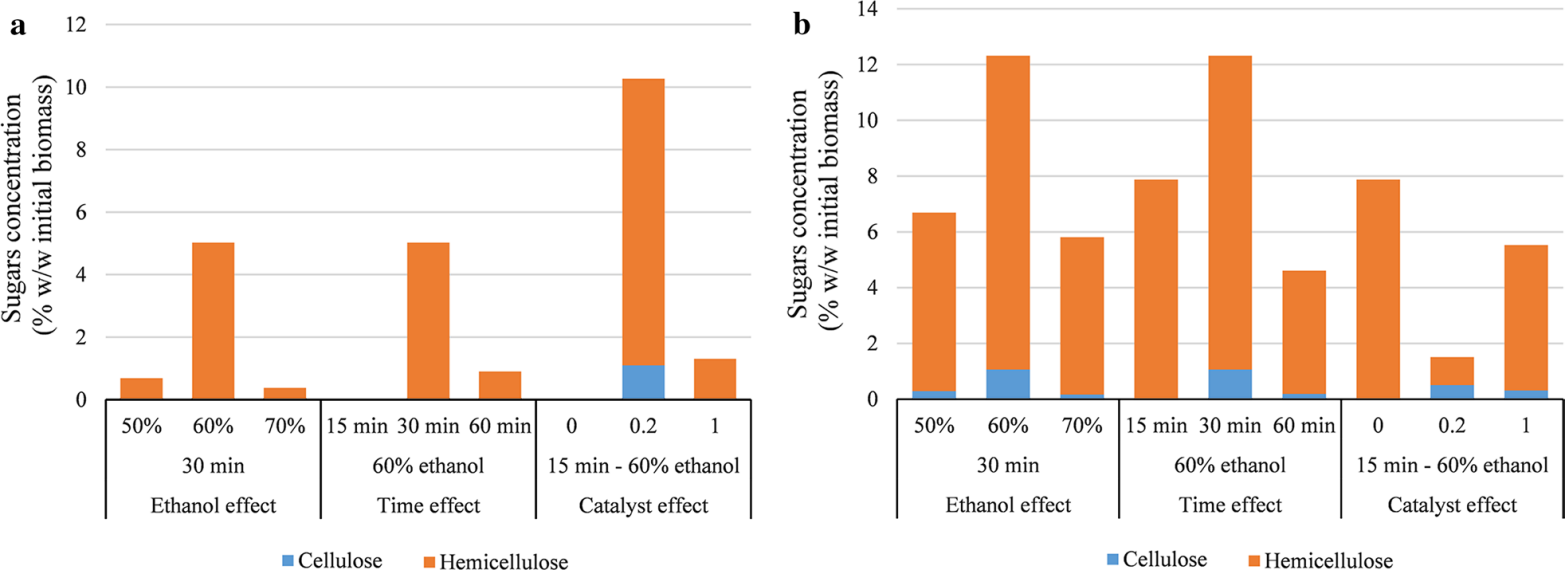
Composition of cellulose and hemicellulose sugars in the liquid fraction after lignin recovery in the form of sugar monomers (**a**) and oligosaccharides (**b**). The analysis was done in duplicates

Figure 2 shows the sugar composition of the aqueous liquid fraction of the pretreated liquor following separation from the solids by vacuum filtration, ethanol evaporation, and lignin recovery. Some of the sugars removed from the biomass during pretreatment were recovered in the pretreatment liquor as a mixture of monomers and oligomers (or soluble polysaccharides). Monomeric sugars of cellulosic origin were relatively low, with glucose detected only in the sample treated with 0.2% acid catalyst; oligomeric glucose accounted for up to 3.1% of the cellulose in untreated biomass. Sugars originating from hemicellulose were more abundant, owing to extensive hemicellulose hydrolysis during pretreatment. The concentration of ethanol used in the pretreatment step affected the total amount of hemicellulosic sugars (both monomeric sugars and oligomers), with the highest concentration obtained with 60% ethanol (Fig. 2). A similar trend was also observed during the pretreatment of wheat straw with acetone–water mixtures, where increasing the solvent content to 40% v/v increased the concentration of hemicellulosic sugars in the liquor, but any further increase had a negative impact on the concentration of the hemicellulosic sugars in the liquor [38]. When treatment time was increased, the concentration of hemicellulosic sugars in the pretreated liquor initially increased, but decreased when the treatment was extended to 60 min, possibly due to the degradation of the monomeric sugars as a result of prolonged heating. This finding correlates well with the results of biomass solubilization (which decreased with prolonged treatment time) and the hemicellulose and lignin contents in pretreated biomass (Table 1). Finally, a 0.2% w/w addition of acid catalyst increased the concentration of sugars in the pretreated liquor (Fig. 2); however, sugar recovery decreased with 1% acid, possibly because of accelerating sugar degradation reactions [7].

Apart from hemicellulosic sugars in the liquor, treatment conditions affected also the ratio between monomeric and oligomeric sugars. In general, sugars found in monomeric form were lower compared to oligomers; only when 0.2% sulfuric acid was used as a catalyst were monomeric sugars approximately nine times more abundant than oligomers. Ethanol content had a minor impact on the ratio between monomeric and oligomeric sugars: it increased initially when ethanol rose from 50 to 60%, but decreased thereafter, probably due to the lower water content and therefore lower generation of hydronium ions that can selectively depolymerize hemicellulose [39]. A short treatment time of 15 min yielded only oligomeric hemicellulosic sugars, whereas a 30-min treatment increased the amount of monomeric sugars in the liquor. Notably, the ratio between monomeric and oligomeric sugars declined when treatment time was prolonged to 60 min, probably due to extended decomposition of hemicellulosic sugars as sugars of hemicellulosic origin (especially xylose) are generally sensitive to thermal degradation under harsher pretreatment conditions [40]. Finally, the addition of acidic catalyst initially increased the ratio of monomeric sugars due to the more acidic conditions created that promoted the depolymerization of oligomeric sugars, whereas, at the highest concentration of 1% w/w, the amount of monomeric sugars was considerably reduced. Recovery of the hemicellulosic sugars in the liquid fraction after the organosolv pretreatment has been found to be significantly dependent on the concentration of the acid catalyst employed as increasing the acid catalyst decreased the recovery [41].

### Evaluation of saccharification efficiency of pretreated solids

Apart from achieving good fractionation yields, one important aspect of establishing a pretreatment process is to produce pretreated solids that present high saccharification yields. High glucose concentration is very important for the subsequent bioconversion processes, such as ethanol fermentation, as it can result in high product titers. Therefore, the first step to assess the potential of the pretreated solids prior to bioconversion is to assess their saccharification yields. For this reason, we performed enzymatic saccharification trials at low solids content aiming to select the materials with high saccharification yields and subsequently evaluate them as raw materials for ethanol production.

**Fig. 3:**
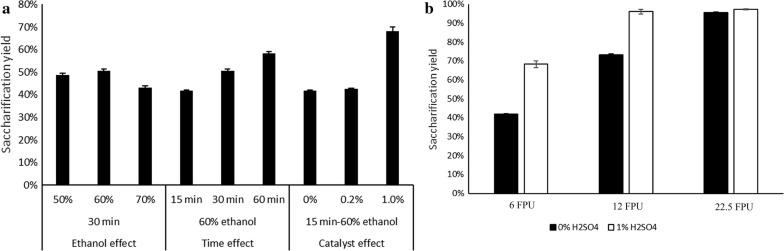
Effects of pretreatment parameters on the enzymatic saccharification of the pretreated solids under a constant enzyme load of 6 FPU/g_solids_ at a solid loading of 2% (**a**). Effects of different enzyme loadings on the saccharification yields with and the without the addition of 1% sulfuric acid for the pretreatment taking place with 60% ethanol for 15 min (**b**). The saccharification yield calculated based on the cellulose content in the pretreated solids

Figure 3a shows the effect of ethanol, treatment time, or acid addition during the pretreatment on the saccharification yields (as defined in Eq. 2) during enzymatic hydrolysis of the pretreated solids. Increasing the acid catalyst concentration had a significant effect on saccharification yield, which increased from 43 to 68%. Instead, increasing treatment time had only a moderate effect, resulting to a saccharification yield of 42, 51, and 58% for 15, 30, and 60 min, respectively, in spite of a reduced delignification yield achieved at 60 min treatment. In contrast, increasing the ethanol concentration to 70% during pretreatment decreased saccharification yield (from 51 to 43%) and delignification (see “Effect on biomass composition and delignification” section). To further examine the effect of acid catalyst addition on saccharification yield, birch biomass treated with and without acid catalyst was tested under various enzyme dosage conditions (Fig. 3b). Presence of acid catalyst during pretreatment increased the saccharification yield at all enzyme loadings tested during the current work. The most profound improvement, however, was observed at the lower enzyme load of 6 FPU/g of enzyme preparation, with an increase in the saccharification yield of 63% compared to birch treated without the addition of acid catalyst. The difference in the saccharification yields between the two pretreated materials decreased with the increasing enzyme dosage, and, indeed, identical yields were reached at the higher enzyme dosage tested, where saccharification was almost complete. Accordingly, addition of acid catalyst enables the use of a lower enzyme dosage. Besides enhanced saccharification yield, catalyst addition resulted in a material with higher cellulose content than when no acid catalyst was added (78% compared to 66%—Table 1), which in turn led to higher glucose concentration in the broth.

**Fig. 4:**
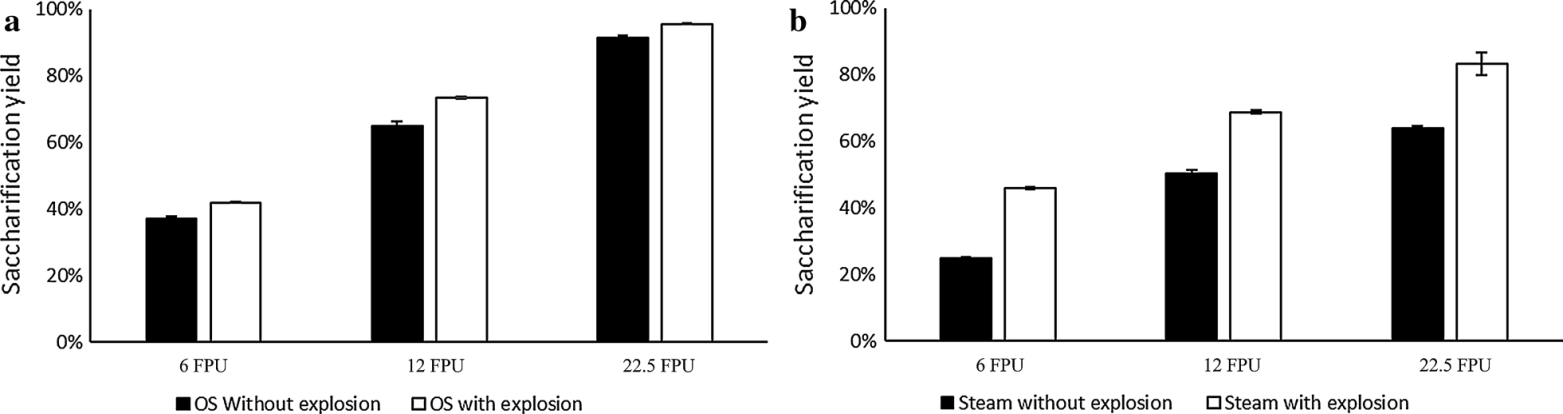
Effect of enzyme dosage on OS (organosolv) samples with and without explosion (**a**); effect of enzyme loading on birch pretreated with steam explosion with and without the explosive discharge (**b**)

The material treated under optimal conditions (60% v/v ethanol, 15 min, 1% w/w H_2_SO_4_) with the newly developed method outperformed the material treated with steam explosion (Figs. 3 and 4). More specifically, the saccharification yields were 68, 97, and 100% for 6, 12, and 22.5 FPU/g enzyme load, respevtively (Fig. 3b). In contrast, the saccharification yields obtained from the material pretreated with steam explosion were 46, 69 and 83%, respectively. Furthermore, due to the difference in cellulose composition, the hybrid pretreated material yielded 590, 830, and 843 mg_glucose_/g for enzyme loadings of 6, 12, and 22.5 FPU/g, respectively. The glucose yields for the steam exploded materials were 292, 437, and 529 mg_glucose_/g, respectively, for the same enzyme loadings. This difference in the glucose concentration is very important for the subsequent microbial conversion processes such as ethanol fermentation.

### Assessing the role of explosive discharge

What differentiates the proposed hybrid solvent organosolv-steam explosion pretreatment approach from more conventional organosolv methods is the combination of solvent cooking with the explosive discharge at the end of pretreatment. This step was applied in an effort to combine the fractionation efficiency of organosolv-type treatments with the positive effect of explosion on enzymatic saccharification, as observed in conventional steam explosion [42]. To evaluate the effect of explosion on enzymatic saccharification of the solids, experiments without explosive discharge were performed (the chosen conditions were 60% v/v ethanol for 15 min without addition of acidic catalyst). The reason to perform the evaluation of the explosive discharge without the use of the acid catalyst was to study the effect of the explosion ‘independently’ without the additive effect of the acid catalyst. Moreover, steam pretreatment experiments with and without explosion (see “Methods” section) were also performed to compare the proposed hybrid process in terms of pretreatment efficiency with a state-of-the-art pretreatment method.

**Fig. 5:**
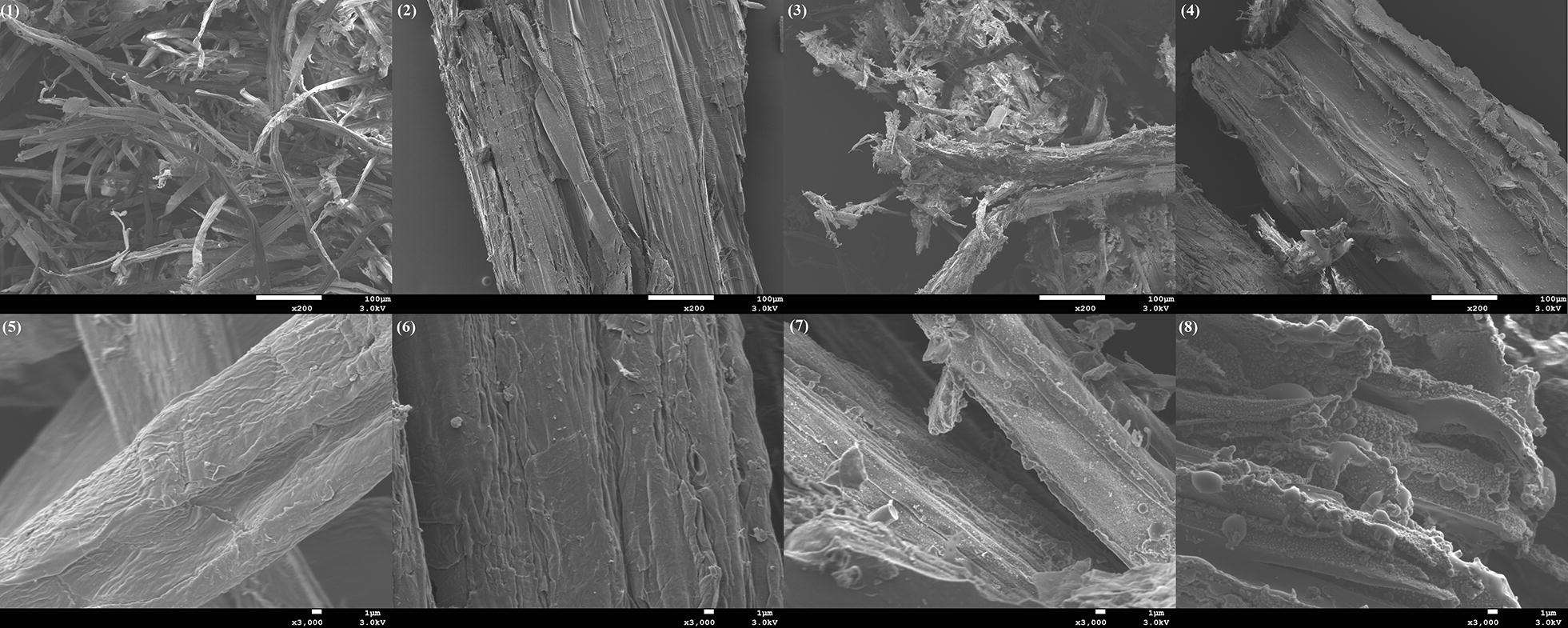
SEM imaging at low (1–4) and high (5–8) magnifications of birch treated with hybrid organosolv pretreatment, with (1, 5) and without (2, 6) the presence of explosive discharge at the end of the pretreatment and treated with traditional steam explosion with (3, 7) and without (4, 8) the presence of explosive discharge at the end of the pretreatment

The chemical composition of pretreatment solids from these experiments is presented in Table 2. For organosolv pretreatment, explosion increased the content of cellulose and hemicellulose on the resulting solid fraction, whereas the content of lignin was slightly decreased. Instead, in steam explosion trials, hemicellulose and lignin were not affected, whereas the non-exploded material presented reduced cellulose content. Next, the effect of the explosive discharge on enzymatic saccharification of treated solids was evaluated for a range of enzyme loadings. Addition of the explosive discharge to traditional organosolv cooking had a positive impact on enzymatic saccharification yield at all tested enzyme loadings (Fig. 4a). This improvement ranged from 4.5 to 13.5%, with the highest gain observed at the lowest enzyme load tested. To better understand the effect of the explosion on enzymatic saccharification of cellulose, the same trial took place with steam explosion pretreatment with or without the explosion step. Again, similar to organosolv treatment, the presence of the explosion at the end of the cooking time significantly improved saccharification yields (Fig. 4b); although the explosion step had a much greater impact compared to the effect on the organosolv. The same positive effect of the explosive discharge on enzymatic saccharification had been reported also for steam explosion-pretreated spruce, corn stover, and beech wood [43, 44]. However, to the best of our knowledge, this is the first time that the effect of the explosive discharge was applied and studied in combination with organosolv cooking. The enzymatic saccharification results were corroborated by observation of the solids’ morphology by SEM (Fig. 5). When the explosive discharge was included at the end of pretreatment, the pretreated fibers appeared smaller and less intact compared to when the explosive discharge was omitted (Fig. 5). Moreover, in the absence of the explosive discharge step, the surface of pretreated fibers seemed smoother and without any significant damage. Similar morphological changes were also observed in the explosive discharge step during the pretreatment of spruce chips [44]. Finally, in contrast to steam explosion, where lignin droplets are deposited on the surface of the biomass, pretreatment with the hybrid process did not result in any lignin deposition (Fig. 5—refer to high magnification images). Although organosolv pretreatment with explosion exhibited only a moderate effect on the saccharification yield compared to the non-exploded, the difference in glucose released per gram of substrate was actually larger owing to a considerably higher cellulose content in the pretreated fibers when explosion was included compared to when it was absent (66% w/w vs 58% w/w).

**Table 2:** Effect of explosive discharge on the composition of pretreated solids

Pretreatment conditions	Cellulose (% w/w)	Hemicellulose (% w/w)	Lignin (% w/w)
Organosolv (200 °C—60% v/v ethanol—15 min)			
With explosion	66.3	22.0	7.8
Without explosion	57.7	17.4	9.1
Steam explosion (200 °C—5 min—0.14% w/w H_2_SO_4_)			
With explosion	57.2	12.1	27.1
Without explosion	46.8	13.0	27.7

Compositional analysis was performed in duplicates

### Ethanol fermentation with pretreated solids

**Fig. 6:**
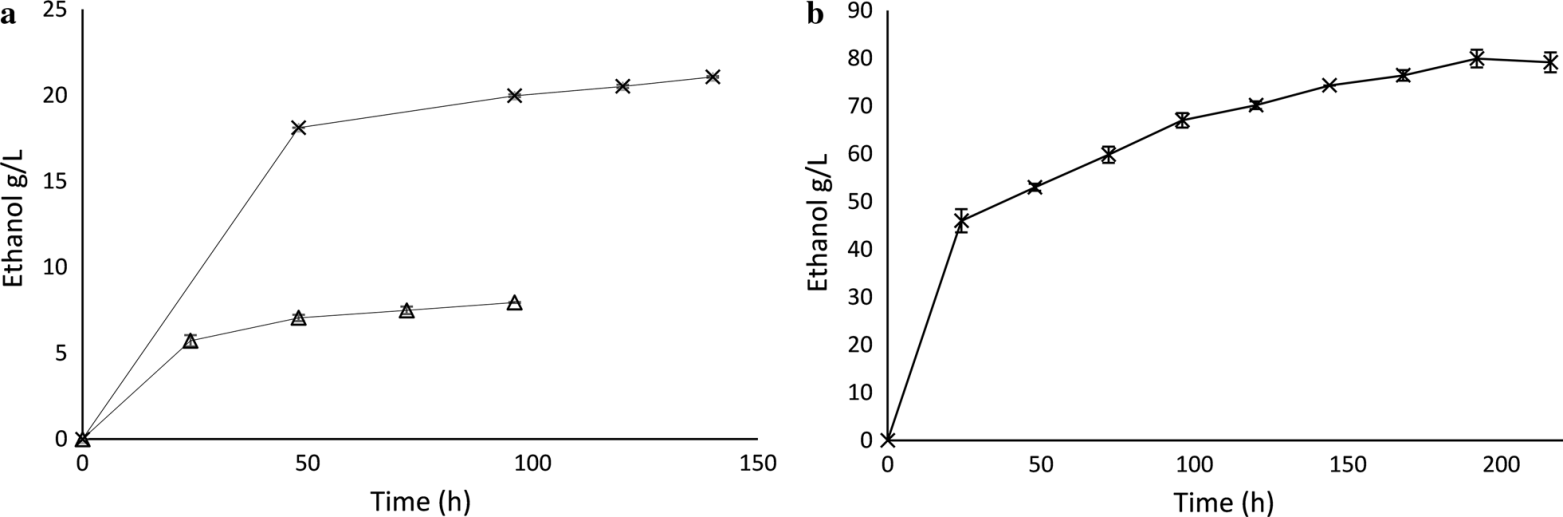
Ethanol profile during SSF with hybrid organosolv-pretreated birch biomass, (**a**) at 5% w/w solids loading with (cross) or without acid (triangle) catalyst, and (**b**) at 20% w/w loading with acid catalyst

Finally, the fermentation potential of pretreated solids was examined under both low- and high-gravity conditions. Initially, fermentability of the pretreated solids was assessed on birch pretreated under the two conditions that yielded the highest saccharification (Fig. 3a): 60% ethanol with 1% acid catalyst for 15 min, and 60% ethanol for 60 min. The ethanol thus obtained reached 21 and 8 g/L, respectively (Fig. 6a), corresponding to yields equal to 100 and 50% of the maximum theoretical value (based on the cellulose content of the pretreated solids). Despite the excellent yields obtained during low-gravity fermentation, the obtained ethanol titers were lower compared to the minimum requirements—40 g/L—for an economically feasible large-scale ethanol distillation [45]. A trend in second-generation ethanol production is to move toward higher gravity to reach more cost effective processes, with higher ethanol titers, better water economy, and more efficient processing [46]. Based on the results obtained from the low-gravity trials, acid-pretreated solids were used for high-gravity fermentation. The initial concentration of solids was rather high, 20% w/w, which posed a challenge for the proper mixing of the material. To overcome this problem, a previously established gravimetric saccharification reactor [47–49] was used for 8 h prehydrolysis. After the prehydrolysis step, the concentration of glucose in the slurry reached 81 g/L, corresponding to a saccharification yield of 47% of the glucan. The high glucose concentration at the beginning of fermentation is a very promising characteristic for the subsequent fermentation step, thereby demonstrating the potential of the pretreated birch biomass. Indeed, ethanol production began rapidly, reaching 46 g/L after only 24 h, with a volumetric productivity of 1.9 g/L h, surpassing the 40 g/L threshold. Thereafter, ethanol productivity diminished compared to the initial 24 h; however, ethanol was produced at a significant rate even after 192 h of fermentation. The highest concentration obtained during this work was 80 g/L, corresponding to a yield of 90% of the theoretical maximum (Fig. 6b). To the best of our knowledge, this is the highest reported ethanol concentration obtained from birch biomass (Table 3), demonstrating the superiority of the proposed hybrid pretreatment method. Also, the ethanol concentration reached in this work was among the highest reported for woody biomass (Table 3). Our novel hybrid solvent organosolv-steam explosion pretreatment method offers a superior solution to the pretreatment of woody biomass, resulting in an inhibitor-free pretreated material that is easily hydrolyzable and yielding high sugar titers at high-solid loadings.

**Table 3:** Ethanol production reported in the literature for high-gravity fermentation of various wood lignocellulosic raw materials

WIS (%)	Material	Pretreatment	Strain	Enzyme loading	Ethanol (g/L)	Time (h)	References
20	Birch	Steam pretreated	KE6-12	20 FPU/g	14.4	144	[50]
20	Spruce	Steam pretreated	Thermosacc Dry	22.5 FPU/g	40	96	[51]
15	Eucalyptus	Organosolv	IR2-9a	20 FPU/g	42	72	[52]
10	Spruce	Steam pretreated	TMB3400	30 FPU/g glucan	45	100	[53]
10	Spruce	Steam pretreated	Ethanol Red	20 FPU/g	45.8	96	[54]
25	Pine	Sulfite	*S. cerevisiae* from Angel Yeast Co. Ltd	15 FPU/g	82	24	[55]
20	Beechwood	Acetone/water oxidation	Ethanol Red	8.4 FPU/g	75.9	120	[56]
20	Eastern red cedar	Acid bisulfite	D_5_A	46 FPU/g glucan	52	42	[57]
20	Birch	Hybrid organosolv—steam explosion	Ethanol Red	18.5 FPU/g	80	192	This study

## Conclusions

Efficient and complete utilization of forest biomass for the production of portfolio of products including renewable fuels such as ethanol, requires the development of a novel biorefinery concept capable of utilizing each biomass component. To achieve this, new fractionation technologies need to be developed. In the present study, we propose a novel hybrid pretreatment/fractionation method that combines the fractionation efficiency of traditional organosolv processes with the explosive discharge at the end of pretreatment. Optimization of pretreatment parameters resulted in 86% delignification and pretreated solids with high cellulose (78%) and low lignin (7%) content. The pretreated solids allowed for high saccharification yields, of up to 68% with low enzyme load and full hydrolysis when enzyme load increased. Finally, use of the pretreated solids as raw material for high-gravity fermentation resulted in an ethanol titer of 80 g/L.

## Methods

### Feedstock

In the present work, wood chips from silver birch (*Betula pendula* L.) originating from mills in Northern Sweden were used. Bark-free chips were air-dried and milled in a Retsch SM 300 knife mill (Retsch GmbH, Haan, Germany) through a 1-mm screen and used for the pretreatment experiments. The composition of untreated birch (expressed in dry basis) was 34.7% w/w cellulose, 31.2% w/w hemicellulose, and 18.7% w/w lignin. The moisture of the chips used during the experiments was 6.0% w/w.

### Pretreatment

**Fig. 7:**
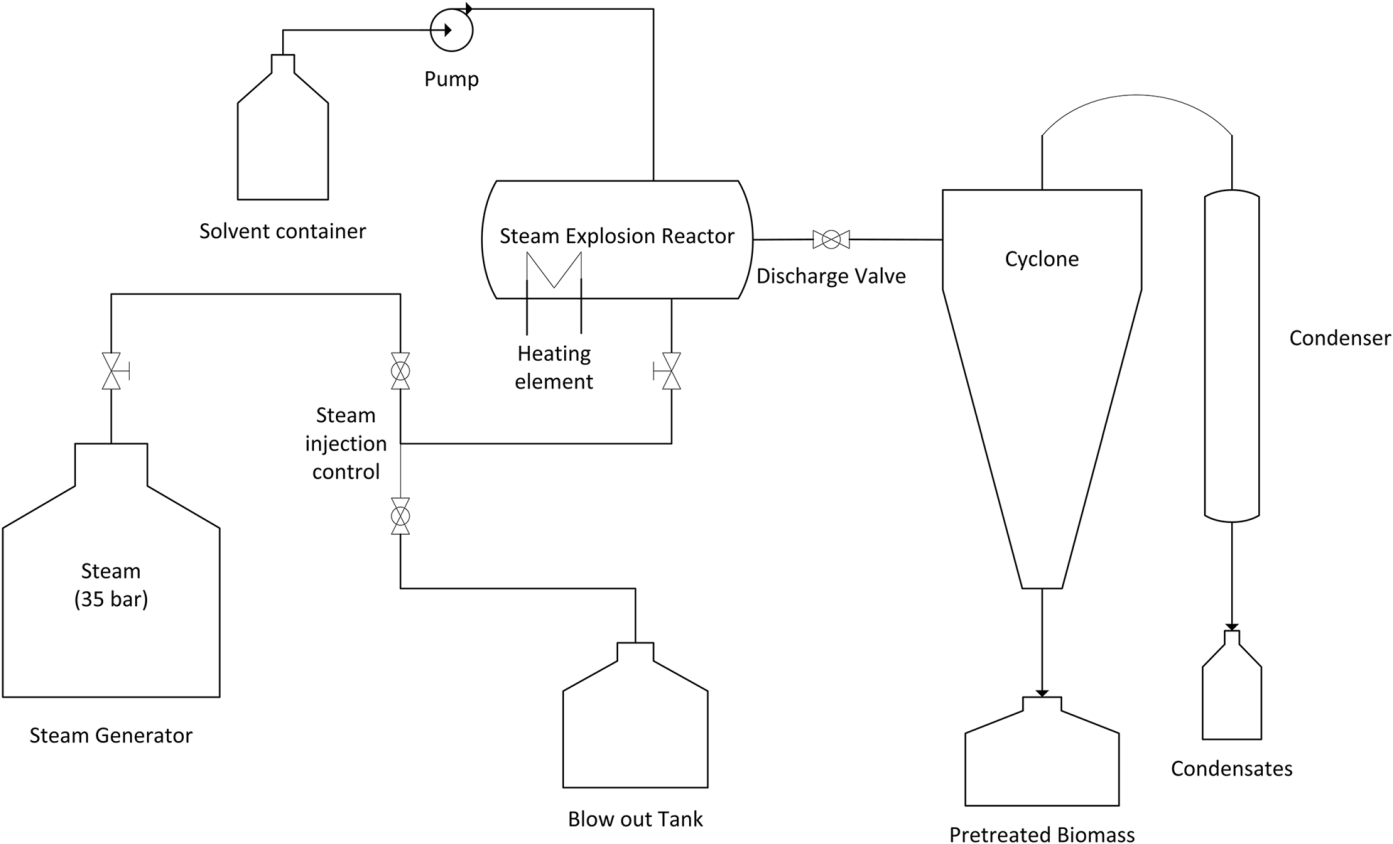
Hybrid solvent organosolv—steam explosion pretreatment and fractionation reactor. The reactor scheme is reprinted from Nitsos et al. [26] under the CC BY-NC-ND 4.0 license

All pretreatment experiments were performed in a horizontal configuration steam explosion reactor modified to operate in organosolv mode [26] (Fig. 7). In short, the apparatus consists of a steam generator, a steam explosion reactor with a discharge valve through which the pretreatment liquor is discharged to a cyclone and finally into a collection vessel, a blowout tank that allows the removal of excess steam condensate generated in the main reactor, and a pump that allows introduction of the organosolv solvent into the reactor. The biomass was manually introduced inside the reactor in a batch mode (200 g of milled birch chips that were mixed with 400 g of ethanol containing the acid catalyst (where applicable), were used per batch of pretreatment). After loading the ‘wet’ biomass, the remainder of the solvent (ethanol) amount was added into the reactor by an external pump to achieve the desired ethanol content of the liquor during pretreatment. Heating of the reactor at the desired temperature was achieved with a combination of steam (internally) and electrical heating elements (externally on the reactor). The introduction of steam as well as the removal of condensate and liquor was controlled by electronically operated valves, except for the discharge valve which was operated manually. At the end of the pretreatment time, the discharge valve was opened, and the pretreatment slurry was exploded through the cyclone and into the collection vessel (see Fig. 7 for a schematic representation of the rector). During the hybrid organosolv-steam explosion trials, the effect of ethanol content (50–70% v/v of the pretreatment liquor), treatment duration (15–60 min), and addition of acid catalyst (sulfuric acid; 0–1% w/w_biomass_) were evaluated under a constant treatment temperature of 200 °C. The solubilization of the main biomass components (cellulose, hemicellulose, and lignin) in the pretreated liquor at the end of the pretreatment was calculated using the following equation

1 $${\text{Component}}\;{\text{solubilization}}\;\left( {\% \;{{\text{w}} \mathord{\left/ {\vphantom {{\text{w}} {\text{w}}}} \right. \kern-0pt} {\text{w}}}} \right) = 100 \times \left( {\frac{{W_{\text{untreat}} \times C_{\text{untreat}} - W_{\text{pretreat}} \times C_{\text{pretreat}} }}{{W_{\text{untreat}} \times C_{\text{untreat}} }}} \right),$$

where *W*_untreat_ and *W*_pretreat_ represent the weights (in grams) of initial untreated solids and of the recovered pretreated solids after the pretreatment, respectively, expressed in dry basis. *C*_untreat_ and *C*_pretreat_ are the contents (% w/w) of the biomass component (cellulose, hemicellulose, or lignin) for the untreated and pretreated biomass, respectively.

Control experiments were performed in the same reactor to determine the effect of solvent pretreatment and of explosive decompression of the pretreatment slurry. These included steam explosion pretreatment, steam non-explosion pretreatment, and organosolv non-explosion pretreatment. Specifically, organosolv non-explosion experiments were performed in the same way as hybrid organosolv-steam explosion with one difference: at the end of the pretreatment time, the discharge valve was not opened. Instead, the valve to the blowout tank was opened, allowing removal of the liquor into the blowout tank and a gradual reduction of pressure; thus avoiding the explosion of the biomass that remained inside the reactor. After removal of the liquid through the valve, the reactor lid was opened, and the pretreated biomass was manually collected from the reactor. The organosolv non-explosion pretreatment was performed at the optimal conditions obtained in the current work, without the addition of the acidic catalyst to better study the effect of the explosion step. Steam explosion experiments (200 °C for 5 min with 0.14% w/w H_2_SO_4_) were performed as previously described [26]. Steam non-explosion experiments were performed by the removal of pretreatment liquid into the blowout tank via the respective valve and the manual collection of biomass from the reactor (as previously described for the organosolv non-explosion pretreatment).

### Lignin recovery

After the organosolv-steam explosion experiment, the pretreatment slurry was vacuum-filtered to separate the solids from the liquor. Ethanol was removed from the liquor in a rotary evaporator to reduce lignin solubility. Lignin was finally isolated by centrifugation (14,000 rpm, 29,416×*g*, at 4 °C for 15 min), air-dried, and analyzed for its purity (composition in carbohydrates and ash—see “Chemical analysis” section). The clear liquor obtained after centrifugation and containing solubilized sugars was collected for sugars determination. Pretreated biomass solids were washed with ethanol to remove surface-bound lignin, air-dried, and stored until further use.

In the steam pretreatment experiments, lignin was not isolated, but the liquid and solid fractions were separated by vacuum filtration, and the solids were washed with deionized water until a neutral pH of the filtrate was achieved.

### Chemical analysis

Analysis of the chemical compositions of untreated and pretreated solid biomass was performed as described elsewhere [58]. Carbohydrates were determined by HPLC analysis employing an Aminex HPX-87P column (BioRad, Hercules, CA, USA), with a column temperature of 85 °C, H_2_O as mobile phase at a flow of 0.6 mL/min, and an RI (refractive index) detector. Acetyl groups were determined by measuring acetic acid with an Aminex HPX-87H column (BioRad, Hercules, CA, USA), 5 mM H_2_SO_4_ as mobile phase at a flow of 0.6 mL/min, and 65 °C. To determine inorganic ash, samples were ashed at 550 °C for 3 h to remove any organic content and the ash was determined gravimetrically. To determine carbohydrate and ash contents in recovered lignin, samples underwent the same procedure as pretreated solid biomass. The same HPLC method as above was used to calculate sugar monomers in pretreatment liquid. To determine sugar oligomers, concentrated sulfuric acid was added to liquid samples to a final concentration of 4%, samples were hydrolyzed at 121 °C for 1 h, and then neutralized, filtrated, and analyzed as described above. Ethanol produced during fermentation was analyzed on an Aminex HPX-87H column using the conditions described before. Biomass moisture content was analyzed with a Sartorius MA 30 (Sartorius AG, Goettingen, Germany) moisture analyzer.

### SEM analysis

Samples were prepared by mounting them on conducting carbon tapes. They were imaged with a scanning electron microscope (JEOL 7800-F Prime) in low vacuum (100 Pa) and high vacuum (10^−4^ Pa or lower), with an acceleration voltage of 3.0 kV. To image the samples in high vacuum, and therefore increase image resolution, a thin coating with palladium was performed. A layer of a few nanometers increased the conductivity sufficiently to image in high vacuum. Images in low vacuum and high vacuum were compared to ensure that the coating did not affect sample morphology.

### Enzymatic hydrolysis trials

Enzymatic hydrolysis tests were performed on pretreated biomass samples to establish their enzymatic saccharification yield and test the efficiency of the pretreatment method. Hydrolysis was performed in cotton-stoppered 100-mL flasks with a dry matter content of 2% (w/v), a final volume of 40 mL, and 50 mM citrate buffer at pH 4.8 using the commercial enzyme solution Cellic^®^ CTec2 (Novozymes A/S, Bagsværd, Denmark) which has an enzyme activity of 149 FPU/g [50]. During the initial trials, we screened the birch biomass obtained using various pretreatment conditions with 6 FPU of enzyme loading per gram of solids. Enzyme dosage tests on the most promising material were carried out with 6, 12, and 22.5 FPU/g solids. Flasks were incubated in a shaken water bath (OLS 200, Grant Instruments, Cambridge, UK), at 120 rpm (orbital arm of 9 mm radius) for 48 h at 50 °C. All experiments were performed in duplicate. Samples obtained before and after hydrolysis (at 0 and 48 h) were filtered through 0.2-µm nylon syringe-filters and stored at − 20 °C until further analysis. Glucose released during hydrolysis was determined using HPLC as described previously [59]. Saccharification yield was defined as

2 $$\eta = 100*\left( {\frac{{C_{\text{glucose}} *V_{\text{liquid}} *0.90}}{{m_{\text{solids}} *x_{\text{cellulose}} }}} \right),$$

where *C*_glucose_ is the concentration of glucose measured by HPLC in g/L, *V*_liquid_ is the volume of the liquid used in the hydrolysis, 0.90 is the correction factor for the addition of a molecule of water during the hydrolytic reaction, *x*_cellulose_ is the mass fraction of cellulose in dry solids, and *m*_solids_ is the mass of the dry solids. The results of the saccharification yield are based on the cellulose content of the pretreated solids.

### Ethanol fermentation trial at low solid concentration

For SSF (simultaneous saccharification and fermentation) at low gravity, samples pretreated with (60% ethanol, 1% H_2_SO_4_, 15 min) and without acid catalyst (60% ethanol, 60 min) were used. As the saccharification yield among non-acid-treated samples was the highest for a 60-min treatment, we used this condition rather than the 15-min one. Biomass was prehydrolyzed for 8 h with 6% w/w solids and then diluted to 5% w/w with the addition of *Saccharomyces cerevisiae* Ethanol Red^®^ and nutrients (1 g/L yeast extract, 0.5 g/L (NH_4_)_2_HPO_4_, and 0.025 g/L MgSO_4_.7H_2_O) for a pitching load of 20 mg dry cell matter/g solids. Samples were taken every 24 h over 5 days for ethanol measurement. They were centrifuged, the supernatant was filtered through a 0.2-µm nylon filter, and analyzed by HPLC as described before. Fermentations were performed in duplicates.

### High-gravity ethanol fermentation

During high-gravity fermentation, birch pretreated for 15 min with 60% ethanol (v/v) and 1% H_2_SO_4_ (w/w) was used. Saccharification took place in a gravimetric saccharification chamber as described previously [47, 48]. Pretreated birch biomass was saccharified at a dry material content of 20% w/w in citrate buffer (50 mM) with 18.5 FPU of Cellic^®^ CTec2 per gram of solids. Saccharification took place at 50 °C for 8 h, after which the slurry was collected and used for SSF. The slurry was supplemented with nutrients to achieve a final concentration of 1 g/L yeast extract, 0.5 g/L (NH_4_)_2_HPO_4_, and 0.025 g/L MgSO_4_·7H_2_O from a concentrated stock solution so that the volume change after addition was < 2% (v/v). The SSF experiment was initiated by addition of *S. cerevisiae* Ethanol Red^®^ suspension (from an overnight YPD culture grown in 250 mL flasks at 35 °C and 180 rpm) amounting to an initial cell concentration of 1 g/L dry cell matter. Samples were taken regularly throughout the cultivations, which were performed in duplicates at 35 °C and 120 rpm. Samples were diluted five times based on mass, filtered through a 0.2-µm nylon filter, and analyzed by HPLC as described in the chemical analysis section.
